# Performance evaluation of newly developed surrogate virus neutralization tests for detecting neutralizing antibodies against SARS-CoV-2

**DOI:** 10.1038/s41598-023-31114-9

**Published:** 2023-03-27

**Authors:** Oh Joo Kweon, Joon-Yong Bae, Yong Kwan Lim, Yoojeong Choi, Sohyun Lee, Man-Seong Park, In Bum Suh, Hana Kim, Young Sam Jee, Mi-Kyung Lee

**Affiliations:** 1 Department of Laboratory Medicine, Chung-Ang University College of Medicine, Seoul, Republic of Korea; 2 Department of Microbiology, Institute for Viral Diseases, Vaccine Innovation Center, College of Medicine, Korea University, Seoul, Republic of Korea; 3 Department of Laboratory Medicine, College of Medicine, Kangwon National University, Chuncheon-si, Republic of Korea

**Keywords:** Immunological techniques, Laboratory techniques and procedures

## Abstract

We evaluated newly developed surrogate virus neutralization tests (sVNT) for detecting neutralizing antibodies (NAbs) against the receptor binding domain of severe acute respiratory syndrome coronavirus 2 (SARS-CoV-2). VERI-Q SARS-CoV-2 Neutralizing Antibody Detection ELISA Kit (MiCo BioMed, Gyeonggi-do, Republic of Korea, hereafter, “eCoV-CN”) is an enzyme-linked immunosorbent assay-based sVNT, and VERI-Q SARS-CoV-2 Neutralizing Antibody Rapid Test Kit (MiCo BioMed, hereafter, “rCoV-RN”) is a point-of-care lateral-flow immunochromatography test with auto-scanner. A total of 411 serum samples were evaluated. Both evaluations used a 50% plaque reduction neutralization test (PRNT_50_) as the gold standard. Compared with PRNT_50_, the eCoV-CN showed 98.7% positive percent agreement (PPA), 96.8% negative percent agreement (NPA), 97.4% total percent agreement (TPA), with kappa values of 0.942. The rCoV-RN showed 98.7% PPA, 97.4% NPA, 97.8% TPA, and kappa values of 0.951, comparing to PRNT_50_. Neither assay indicated cross-reactivity for other pathogens, and the signal indexes were statistically significantly correlated to the PRNT_50_ titer. The two evaluated sVNTs show comparable performances to the PRNT_50_ with the advantages of technical simplicity, speed, and do not require cell culture facilities.

## Introduction

Humoral immunity to severe acute respiratory syndrome coronavirus 2 (SARS-CoV-2) induced either through natural infection or vaccination has been shown to reduce the risk of clinically significant outcomes and/or afford a degree of protection against reinfection^1,2^. For SARS-CoV-2, neutralizing antibodies (NAbs) that bind to the receptor binding domain (RBD) of spike (S) protein have the potential to neutralize viral entry into cells and are thought to play an important role in the protective immune response to SARS-CoV-2 infection^3^. Furthermore, access to NAbs would help determine the immunity of a community against SARS-CoV-2.

The current reference standard for detecting NAbs is the virus neutralization test (VNT), which can be a plaque reduction neutralization test (PRNT)^4–7^. However, this culture-based test requires live viruses and a biosafety level 3 containment facility, highly skilled operators, and is too cumbersome to be routinely performed^7^.

To overcome these problems, surrogate VNTs (sVNTs) for detecting NAbs against the RBD of SARS-CoV-2 have been developed. The sVNTs are independent of the use of living or pseudotyped viruses and cell cultures, allowing for high-throughput, automation, and fast turnaround time. VERI-Q SARS-CoV-2 Neutralizing Antibody Detection ELISA Kit (MiCo BioMed, Gyeonggi-do, Republic of Korea, hereafter, “eCoV-CN”) is an enzyme-linked immunosorbent assay (ELISA) for SARS-CoV-2 NAbs detection, and VERI-Q SARS-CoV-2 Neutralizing Antibody Rapid Test Kit (MiCo BioMed, hereafter, “rCoV-RN”) is a point-of-care immunochromatographic immunoassay. These assays rely on competitive inhibition of NAbs in the interaction of ACE-2 protein with enzyme-labeled and purified RBD from S protein in the same manner as in classical VNTs. In this study, we evaluate the analytical performances of two newly developed sVNTs for detecting NAbs using convalescent sera of COVID-19 patients compared to the 50% PRNT (PRNT_50_).

## Results

### Cut-off establishment and semi-quantitative correlation analysis

**Figure 1:**
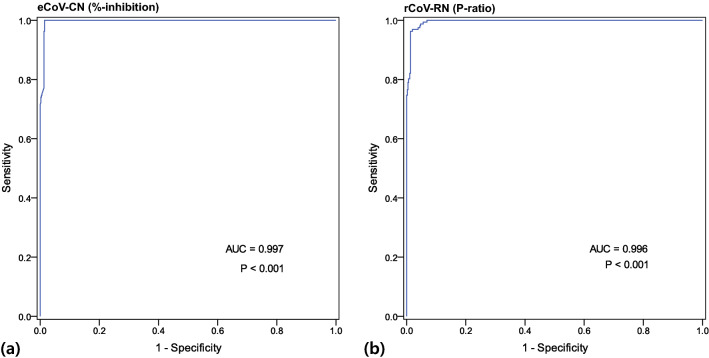
Receiver-Operating Characteristic analysis for detecting NAbs of SARS-CoV-2 of (**a**) VERI-Q SARS-CoV-2 Neutralizing Antibody Detection ELISA Kit (eCoV-CN) and (**b**) VERI-Q SARS-CoV-2 Neutralizing Antibody Rapid Test Kit (rCoV-RN). The optimal cut-off values for %-inhibition of eCoV-CN and *P* ratio of rCoV-RN obtained from the analysis were 30% and 0.83, respectively.

The receiver operating characteristics (ROC) curve analysis to determine the cut-off for eCoV-CN and rCoV-RN are illustrated in Fig. 1. For eCoV-CN, 30% was determined as the cut-off for the presence of NAbs, with the sensitivity and specificity of 96.3% and 98.7%, respectively (Youden index J was 0.950), with the area under the ROC curve (AUC) value of 0.997 (95% confidence level 0.998–1.000, *P* < 0.0001). For rCoV-RN, ROC curve analysis revealed that ≥ 0.83 of the *P* ratio (signal value ratio generated from the rCoV-RN, details are described below “Methods” section below) was the best cut-off for the positive for NAbs. At the 0.83 cut-off, the sensitivity and specificity were 96.30% and 98.72% (Youden index J was 0.950), respectively with the AUC value of 0.996 (95% CI 0.987–0.999, *P* < 0.0001).

**Figure 2:**
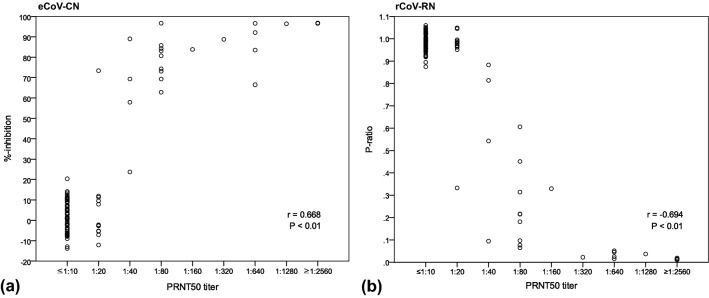
Correlation analysis between PRNT_50_ titer and (**a**) %-inhibition values from VERI-Q SARS-CoV-2 Neutralizing Antibody Detection ELISA Kit (eCoV-CN) and (**b**) *P*-ratio from VERI-Q SARS-CoV-2 Neutralizing Antibody Rapid Test Kit (rCoV-RN), conducted using 105 sera, including 30 from the patients infected with SARS-CoV-2.

Correlation analysis between PRNT_50_ titer and %-inhibition or *P*-ratio obtained from the evaluated sVNTs is illustrated in Fig. 2. Both assays showed statistically significant positive or negative Spearman’s rho (r) values to the PRNT_50_ (0.668 for eCoV-CN and − 0.694 for rCoV-RN, both *P* < 0.01).

### Diagnostic accuracy and cross-reactivity

The diagnostic accuracy of eCoV-CN compared to PRNT_50_ is listed in Table 1. Positive percent agreement (PPA) and negative percent agreement (NPA) of eCoV-CN to the PRNT_50_ were 98.7% and 96.8%, respectively, with the Kappa value of 0.942 (almost perfect agreement). The total percent agreement (TPA) between the two assays was 97.4%.

**Table 1:** Diagnostic accuracy of VERI-Q SARS-CoV-2 Neutralizing Detection ELISA Kit for the detection of neutralizing antibodies against SARS-CoV-2 compared to the 50% plaque reduction neutralization test.

VERI-Q SARS-CoV-2 Neutralizing Detection ELISA Kit	PRNT_50_		PPA, % (95% CI)	NPA, % (95% CI)	TPA, % (95% CI)	Kappa (95% CI)
	Positive	Negative				
Positive	74	5	98.7 (92.8–100)	96.8 (92.7–99.0)	97.4 (94.3–98.9)	0.942 (0.895–0.988)
Negative	1	151				

*PPA* positive percent agreement; *NPA* negative percent agreement; *TPA* total percent agreement; *PRNT* plaque reduction neutralization test.

The diagnostic accuracy of rCoV-RN is listed in Table 2. PPA and NPA of rCoV-RN were 98.7% and 97.4%. The total agreement and kappa values between the two assays were 97.8% and 0.951 (almost perfect agreement), respectively. The PPA, NPA, TPA, and Kappa values were identical regardless of interpreting the results of the rCoV-RN by visual inspection or using an auto-scanner.

**Table 2:** Diagnostic accuracy of VERI-Q SARS-CoV-2 Neutralizing Detection ELISA Kit for the detection of neutralizing antibodies against SARS-CoV-2 compared to the 50% plaque reduction neutralization test.

VERI-Q SARS-CoV-2 Neutralizing Rapid Test Kit	PRNT_50_		PPA, % (95% CI)	NPA, % (95% CI)	TPA, % (95% CI)	Kappa (95% CI)
	Positive	Negative				
Visual inspection						
Positive	74	4	98.7 (92.8–100)	97.4 (93.6–99.3)	97.8 (94.9–99.2)	0.951 (0.909–0.993)
Negative	1	152				
Auto-Scanner						
Positive	74	4	98.7 (92.8–100)	97.4 (93.6–99.3)	97.8 (94.9–99.2)	0.951 (0.909–0.993)
Negative	1	152				

*PPA* positive percent agreement; *NPA* negative percent agreement; *TPA* total percent agreement; *PRNT* plaque reduction neutralization test.

Neither eCoV-CN or rCoV-RN cross-reacted (0%, 0/75) to the 75 samples obtained from patients with human immunodeficiency virus, human coronaviruses (HCoVs) other than SARS-CoV-2 (HCoV-NL63, -229E, -HKU, and -OC43), *Haemophilus influenzae,* respiratory syncytial virus, influenza A/B virus, or hepatitis C virus.

## Discussion

This study demonstrates the “almost perfect” concordance between the evaluated sVNTs and the gold standard PRNT_50_ for SARS-CoV-2 NAb detection in human sera. For eCoV-CN and rCoV-RN, the TPA to the PRNT_50_ is 97.4% and 97.8%, respectively. The assays do not cross-react with other HCoVs (including NL63, 229E, HKU and OC43), and signal indexes (%-inhibition and *P*-ratio, respectively) significantly correlate to the semi-quantitative PRNT_50_ titers.

There were three types of neutralization tests used to determine an antibodies’ functional ability to prevent SARS-CoV-2 infection in vitro. SARS-CoV-2 or recombinant SARS-CoV-2 expressing reporter proteins were used in VNT, such as PRNT and microneutralization. These tests were cell culture-based, and plaque formation is observed after incubation; thus, it may take up to 5 days to complete and requires a BSL-3 laboratory to perform safely; therefore, the majority of laboratories are unable to perform them. Another assay used was the pseudovirus neutralization test (pVNT), which used recombinant pseudoviruses that incorporate the S protein of SARS-CoV-2. This assay can be safely performed in BSL-2 laboratories, and neutralization tests are performed in a similar manner in the classic plaque-reduction format. However, measuring neutralizing activity using recombinant pseudoviruses was also challenging. The engineered chimeric strain or pseudovirus requires optimization with regard to surface protein density and structure to ensure that the chimeric constructs correctly mimic the interaction between the native virus and its target and retain the original infectivity. This step is complex and labor-intensive in the development stage, and moreover, the pVNT will require 3 to 5 days to obtain the results because it is also a culture-based test^8,9^. To overcome the disadvantages of classical VNT or pVNT, the sVNT or competitive neutralization tests were developed as simple and rapid assays in conventional competitive immunoassay formats.

In this study, we evaluate assays which use receptor binding domain (RBD) of S1 protein for NAbs detection. However not all NAbs are necessarily RBD-binding antibodies; other regions in the S1 or S2 protein can also play a role in virus neutralization, indicated by past studies with SARS-CoV^10,11^. However, RBD-targeting NAbs are immunodominant during SARS-CoV-2 infection^12^, and a previous study demonstrated that the RBD protein performed better than the S1 protein for NAbs detection^10^.

One of the drawbacks of the evaluated test is that they cannot differentiate whether the NAbs are induced from a past infection or the vaccination because those assays target only the RBD of S proteins. To overcome such drawbacks, a serologic assay targeting the nucleocapsid (N) protein of SARS-CoV-2 should be conducted alongside these assays. Most vaccines targeted the S protein to generate a immune response; thus the presence of anti-N antibodies reflected past infection^13^.

To assess NAbs against SARS-CoV-2, sVNTs and classical competitive indirect ELISAs targeting anti-SARS-CoV-2 RBD antibodies are also widely used. Previous studies revealed that signal indexes (such as optical density or %-inhibition values) from indirect ELISAs and sVNTs correlated well to classical VNT or pVNT titer results^14,15^. However, sVNTs reflect the actual neutralizing potency of NAbs between the virus and host cell components (RBD and ACE-2), not just the presence of RBD-specific antibodies as shown by the indirect ELISA. Another key advantage of sVNTs over ELISAs is the ability to detect total NAbs in an isotype-independent manner^10^, simplifying the test strategy and further increasing the test sensitivity compared to isotype-specific ELISAs (especially, IgG-specific ELISAs). However, the exact mechanism remains unclear; neutralization synergy effects of different isotype antibodies targeting different neutralization-critical epitopes are one of the possible causes^10,16^.

The two assays we evaluate do not offer the cut-off values for the presence of NAbs, therefore we determine these by conducting PRNT tests. The %-inhibition cut-off values of the ELISA format of cVNTs for SARS-CoV-2 differ according to the assays. In this study, we determine a 30% cut-off as its ideal eCoV-CN cut-off. For the rCoV-RN, the lateral flow immunoassay, the auto-scanner value for *P*-ratio is 0.83. By visual inspection, it may be difficult to interpret test results to compare the intensity of the T and C lines. Thus, inexperienced operators are recommended to use a read-out device or auto-scanner.

This study has several limitations. First, we do not assess the clinical conditions or characteristics of the patients. Second, the cross-reactivity of the assay was not fully investigated with the sera from the patients that have been previously infected with other respiratory pathogens than respiratory syncytial virus, influenza virus, and H. influenzae. The cross-reactivity tests to the SARS-CoV and the middle east respiratory syndrome coronavirus were also not conducted. Finally, we cannot assess the semiquantitative correlation analysis between the tested assays.

In conclusion, the two evaluated sVNTs, VERI-Q SARS-CoV-2 Neutralizing Antibody Detection ELISA Kit and VERI-Q SARS-CoV-2 Neutralizing Antibody Rapid Test Kit, show comparable performances to the PRNT_50_. The sVNTs have the advantage of technical simplicity, speed, and cell culture facilities are unnecessary. Therefore, sVNTs would be useful tools for laboratories to assess the humoral immunity or NAbs against SARS-CoV-2 infection, as an alternative to the culture-based VNTs.

## Material and methods

### Study design

This work was performed in two tertiary hospitals, Chung-Ang University Hospital in Seoul and Kangwon National University Hospital in Chuncheon, the Republic of Korea, from March 2021 to December 2021.

This study was conducted in two main steps; i) cut-off establishment and semi-quantitative correlation analysis for each assay to determine the presence of NAbs compared to the results of PRNT_50_ and ii) qualitative diagnostic accuracy evaluation of each assay, including PPA or clinical sensitivity and NPA or specificity. Both evaluation steps were conducted using PRNT_50_ as the gold standard method for the NAb assay^5,6^. A cross-reactivity study for pathogens other than SARS-CoV-2 was also performed.

### Clinical samples

To establish the signal index cut-off value for sVNTs evaluated in this study, 105 serum samples were used. A correlation analysis between PRNT_50_ titer and %-inhibition or *P*-ratio obtained from the evaluated sVNTs was also conducted. Among 105 samples, 30 were obtained from patients suffering or recovering from COVID-19. Others were obtained from the Chung-Ang University Hospital Human Biobank (Seoul, Republic of Korea), which had been collected before the emergence of SARS-CoV-2 in December 2019.

For the performance evaluation of each assay, a total of 231 serum samples were used. All serum samples were obtained from the subjects not vaccinated for SARS-CoV-2. Among the retrospective specimens confirmed to be infected with COVID-19 using STANDARD™ M nCoV Real-Time Detection kit or Allplex™ 2019-nCoV Assay kit, retrospective samples confirmed as positive or negative for neutralizing antibodies by the PRNT method were used. In addition, these samples were from individuals who were unvaccinated against COVID-19.

For the cross-reactivity test, another 75 samples were used. The samples had been collected from patients infected with HIV (N = 10), HCoVs other than SARS-CoV-2 (including HCoV-NL63, -229E, -HKU and -OC43, N = 20), positive for *Haemophilus influenzae* (N = 10), respiratory syncytial virus (N = 10), influenza A/B virus (N = 20), or hepatitis C virus (N = 5).

### NAb assays

#### Enzyme-linked immunosorbent assay

VERI-Q SARS-CoV-2 Neutralizing Antibody Detection ELISA Kit (eCoV-CN) is an sVNT in ELISA format for detecting SARS-CoV-2 specific NAbs in serum. The assay was performed manually according to the manufacturer’s instructions. Briefly speaking, positive/negative controls and samples were mixed 1:2000 with diluted RBD-horseradish peroxidase solution and incubated for 30 min at 37ºC. Next, 100 μL of controls and samples were loaded in 96 microplate wells pre-coated with the angiotensin-converting enzyme-2 (ACE2) in duplicates. After a 15 min incubation at 37ºC and washing, 100 μL of tetramethylbenzidine solution was added to each well. After another 15 min incubation at room temperature, 50 μL of stop solution was added. After adding the stop solution, the optical density (O.D) or absorbance of the resulting product was measured by the Synergy HT (BioTek, Winooski, VT) at the wavelength of 450 nm. For the valid results, the O.D of positive and negative control was required to be < 0.1 and > 1.0, respectively. The inhibition percent of each sample was calculated as “inhibition percent (%-inhibition) = [1-(sample mean O.D / negative control mean O.D)] × 100”.

#### Immunochromatographic assay

VERI-Q SARS-CoV-2 Neutralizing Antibody Rapid Test Kit (rCoV-RN) is a sVNT in lateral flow immunochromatographic assay format for detecting SARS-CoV-2 specific NAbs in serum. It is point-of-care rapid testing that can generate the results within 30 min. This assay consists of a gold pad, ACE2-Fc pad, C line, and T line. The gold pad contains chicken IgY, and RBD conjugated with gold nanoparticles (GNP). The ACE2-Fc pad contains ACE2 conjugated with mouse Fc subunit. T and C lines have goat anti-mouse IgG and anti-chicken IgY, respectively. A total of 10 μL of serum samples and 3 drops of buffer solution were loaded into the sample loading well. If the samples were positive for NAbs, it formed a complex between GNP-RBD-NAbs and GNP-chicken IgY on the gold pad. Then, GNP-RBD-NAbs could not react to ACE2-Fc on the ACE2-Fc pad, and did not generate a visible line in the T line of anti-mouse IgG, and only the C line would change to visible because GNP-chicken IgY can react to goat anti-chicken IgY in the C line; thus, NAbs positive samples had higher color intensity in the C line than T line. In contrast, NAb negative samples cannot generate GNP-RBD-NAbs in the gold pad; only GNP-RBD and GNP-chicken IgY complexes are formed. Then in ACE2-Fc pad, GNP-RBD can be bound to the ACE2-Fc. Thus GNP-RBD-ACE2-Fc complexes are formed. This molecule could react to the goat anti-mouse IgG on the T line; thus, T line color changes occurred. The C line may also be visible because of the GNP-chicken IgY complex. Interpretation of the results can be conducted by visual inspection and auto-scanner Veri-Q PinoView (MiCo BioMed). Visual inspection of the tested sVNT, rCoV-RN, was performed using “color scale (reference pictures),” provided by the manufacturer; the overall intensity of the T and C lines were scored ranging from 5 for strong intensity to 1 for very low/no intensity. A NAb positive sample produced a less intense or lighter color intensity in the T line than the C line. Visual inspection was conducted in a blinded manner, without knowing the auto-scanner results, by a technician other than the auto-scanner operator. Auto-scanner can generate the *P* ratio, which is calculated by the signal value of the T line / C line. This *P* ratio was used as an index for the cut-off value determination study. Visual inspection and auto-scanner were used for the performance evaluation, and their performances were calculated separately.

#### Plaque reduction neutralization test

The SARS-CoV-2 (BetaCoV/Korea/KCDC03/2020, S clade, National Culture Collection for Pathogens [NCCP] 43326) was used to assess the NAb titer. During the period of serum sample collection, GH clade (B.1.497) was the dominant strain in the Republic of Korea^17^. When comparing the RBDs of GH clade and S clade, there were no differences in composition^18^. Therefore, we proceeded to conduct PRNT_50_ with the S clade.

Serum samples were serially diluted two-fold and mixed with equal amounts of virus containing 100 plaque-forming units at 37 °C for 1 h (h). The virus–serum mixtures were inoculated into Vero cells to measure the PRNT_50_. The PRNT_50_ titer was calculated as the highest serum dilution that showed a 50% reduction in the number of viral plaques in comparison with that of a PBS-treated control. The PRNT_50_ cut-off value for the presence of NAbs was determined as higher than 1:20 based on the previous study^19^ and our results from 75 serum samples collected before the COVID-19 pandemic, December 2019 (Supplement Table 1).

### Statistics

For the cut-off establishment study, the ROC curve analysis was performed using MedCalc version 20.014 (MedCalc Software Ltd, Ostend, Belgium). For ROC curve analysis, samples were analyzed using three different kit lots, each in duplicates; thus, 6 results were generated from the evaluated assays. The ROC curve analysis calculated the AUC of each assay and Youden index J at the ideal cut-off point.

For the correlation analysis, the Spearman’s rank test was conducted using IBM SPSS statistics version 20 (IBM, Armonk, NY). Diagnostic accuracy, including PPA and NPA, was calculated for performance evaluation using Microsoft Excel 2016 (Microsoft, Redmond, WA).

### Ethic statements

The study protocol was approved by the institutional review board (IRB) of Chung-Ang University Hospital (Seoul, Republic of Korea; approval no. 2111-020-482) and Kangwon National University Hospital (Chuncheon-si, Republic of Korea; approval no. KNUH-2021-09-018/KNUH-2021-09-022). Obtaining informed consent was waived according to the Chung-Ang University Hospital and Kangwon National University Hospital IRBs’ policy. All experiments were performed in accordance with relevant guidelines and regulations. This research had been performed in accordance with the Declaration of Helsinki.

## Supplementary Information

Supplementary Table 1.

## Data Availability

The datasets used and/or analyzed during the current study available from the corresponding author on reasonable request.
