# Supplementary material for: Performance evaluation of newly developed surrogate virus neutralization tests for detecting neutralizing antibodies against SARS-CoV-2
Source: Sci Rep. 2023 Mar 27;13:4961. doi: 10.1038/s41598-023-31114-9 (PMC10041486; doi:10.1038/s41598-023-31114-9)
Supplement: Supplementary file 1 — Supplementary Table 1. [file 41598_2023_31114_MOESM1_ESM.docx]

**Supplementary Table 1.** Plaque reduction neutralization test (PRNT) results for determining positive titer cut-off values of PRNT_50_ using 75 serum samples collected before December 2019(prior to the SARS-CoV-2 emergence).

|  | Plaque Formation Unit | | |
| --- | --- | --- | --- |
| Sample Dilution Ratio in PRNT | 1:10 | 1:20 | 1:40 |
| Sample No. |  |  |  |
| 1 | 1 | 27 | 84 |
| 2 | 57 | 84 | 94 |
| 3 | 36 | 79 | 85 |
| 4 | 30 | 60 | 90 |
| 5 | 44 | 75 | 83 |
| 6 | 40 | 63 | 73 |
| 7 | 39 | 48 | 79 |
| 8 | 47 | 78 | 95 |
| 9 | 56 | 71 | 94 |
| 10 | 72 | 100 | 101 |
| 11 | 67 | 97 | 103 |
| 12 | 60 | 86 | 113 |
| 13 | 62 | 95 | 117 |
| 14 | 61 | 92 | 106 |
| 15 | 59 | 82 | 90 |
| 16 | 59 | 74 | 101 |
| 17 | 52 | 81 | 88 |
| 18 | 30 | 56 | 92 |
| 19 | 42 | 69 | 76 |
| 20 | 39 | 58 | 93 |
| 21 | 31 | 60 | 84 |
| 22 | 45 | 86 | 105 |
| 23 | 33 | 75 | 105 |
| 24 | 47 | 68 | 90 |
| 25 | 38 | 51 | 85 |
| 26 | 45 | 85 | 80 |
| 27 | 45 | 70 | 85 |
| 28 | 30 | 72 | 88 |
| 29 | 40 | 60 | 88 |
| 30 | 45 | 68 | 80 |
| 31 | 47 | 66 | 107 |
| 32 | 40 | 78 | 107 |
| 33 | 36 | 97 | 92 |
| 34 | 74 | 91 | 112 |
| 35 | 59 | 69 | 105 |
| 36 | 32 | 55 | 70 |
| 37 | 56 | 70 | 95 |
| 38 | 36 | 51 | 77 |
| 39 | 45 | 84 | 93 |
| 40 | 45 | 68 | 90 |
| 41 | 54 | 86 | 97 |
| 42 | 37 | 53 | 82 |
| 43 | 63 | 73 | 91 |
| 44 | 44 | 90 | 95 |
| 45 | 46 | 81 | 96 |
| 46 | 42 | 70 | 77 |
| 47 | 68 | 79 | 101 |
| 48 | 45 | 76 | 90 |
| 49 | 52 | 84 | 112 |
| 50 | 42 | 74 | 92 |
| 51 | 61 | 86 | 100 |
| 52 | 36 | 60 | 86 |
| 53 | 38 | 70 | 77 |
| 54 | 31 | 45 | 70 |
| 55 | 31 | 72 | 82 |
| 56 | 63 | 80 | 100 |
| 57 | 39 | 76 | 99 |
| 58 | 24 | 59 | 77 |
| 59 | 58 | 64 | 88 |
| 60 | 36 | 61 | 90 |
| 61 | 26 | 65 | 91 |
| 62 | 40 | 93 | 104 |
| 63 | 61 | 94 | 100 |
| 64 | 52 | 99 | 97 |
| 65 | 40 | 80 | 82 |
| 66 | 41 | 67 | 120 |
| 67 | 39 | 80 | 110 |
| 68 | 40 | 68 | 90 |
| 69 | 27 | 78 | 91 |
| 70 | 22 | 52 | 79 |
| 71 | 33 | 56 | 75 |
| 72 | 51 | 76 | 97 |
| 73 | 44 | 79 | 96 |
| 74 | 36 | 62 | 109 |
| 75 | 51 | 107 | 105 |
